# Bisphenol A and human fertility: a systematic review

**DOI:** 10.5935/1518-0557.20250029

**Published:** 2025

**Authors:** Carolina Paz Mohamad Isa, Gabriela Barella Schmidt, Renata Guerreiro de Jesus, Joselaine Sturmer, Júlia Prauchner de Castilhos, Laura Randon Chapochnicoff, Victória Campos Dornelles, Marta Ribeiro Hentschke, Alvaro Petracco, Mariangela Badalotti

**Affiliations:** 1 Pontificia Universidade Católica do Rio Grande do Sul, Porto Alegre, Brazil; 2 Federal University of Rio Grande do Sul, Porto Alegre, Brazil; 3 Fertilitat - Medical Reproductive Center, Porto Alegre, Brazil

**Keywords:** bisphenol A (BPA), human fertility, endocrine disruptors, assisted reproduction, sperm production

## Abstract

Considering the significant exposure to the synthetic compound bisphenol A (BPA), present in a wide range of materials in our daily lives, this article discusses a possible correlation between this substance and human fertility through a bibliographic review. In the context of growing evidence that BPA impacts the fertility of women and men of reproductive age, the reviewed articles suggest that exposure to this agent may affect ovarian reserve parameters in women. In pregnant women, it may cause fetal malformations. BPA has also been linked to an increase in spontaneous abortions and premature births. Additionally, it can cause hormonal disruptions, affect folliculogenesis, and worsen ovarian response in assisted reproduction, as well as lead to lower estradiol concentrations, reduced fertilization rates, and higher implantation failure. In men of reproductive age, BPA may decrease sperm production, potentially contributing to testicular dysgenesis syndrome and cryptorchidism. However, further studies are still required to better understand the diverse and complex mechanisms through which BPA affects key reproductive functions.

## INTRODUCTION

Bisphenol A (BPA) is a synthetic compound consisting of two phenolic rings connected by a methyl bridge, and it is used in a wide range of everyday products, including general packaging, DVDs, computers, electronic devices, coatings for food and beverage cans, toys, dental materials, disposable cups and cutlery, medical equipment, thermal paper, and products for children and babies. It is widely used in the production of polycarbonate plastics and epoxy resins. Human exposure to this substance is so extensive that contact can be considered continuous (Konieczna *et al.*, 2015; Pallotti *et al.*, 2020). Exposure to BPA occurs through oral, inhalation, and transdermal routes, via the objects in which it is present.

Studies have demonstrated that BPA interacts with estrogen receptors and can act as either an agonist or antagonist through endocrine receptor-dependent signaling pathways (Konieczna *et al.*, 2015). This interaction can lead to hormonal changes, consequently suggesting the risk of ovarian cysts and breast cancer (Konieczna *et al.*, 2015).

There is growing evidence that BPA impacts both female and male fertility. In women of reproductive age, the exposure to BPA may affect ovarian reserve parameters (Czubacka *et al.*, 2021). In pregnant women, BPA may cause fetal malformations and even miscarriage. In man, BPA may reduce sperm production, potentially leading to testicular dysgenesis syndrome and cryptorchidism (Matuszczak *et al.*, 2019). However, further human studies are needed to better understand its long-term consequences, particularly on offspring.

Thus, the objective of this present study was to review the currently available literature on this subject.

## METHODS

This article is based on bibliographic research and a descriptive-documentary study, following the format of a systematic review. An advanced search was conducted on the Public Medline database platform (PubMed), Scientific Electronic Library Online (SciELO), and Latin American and Caribbean Health Sciences Literature (LILACS), and was performed in September 2024. The database search used the keywords ‘infertility’ and ‘bisphenol A,’ applying the Boolean operator ‘AND.’ Scientific articles discussing the impact of bisphenol A on male and female fertility were included in the study, focusing on hormonal dysregulation affecting the hormonal cycle, gamete quality, fertilization problems, and subsequent issues in pregnancy.

Articles published in English from 2012 onward were selected. The study excluded case reports, book chapters, letters to the editor, viewpoints, studies with hypothetical data or scenario simulations, and studies that did not meet the inclusion criteria. This search yielded a total of 158 studies.

Each manuscript was evaluated based on its title and abstract, according to the inclusion criteria. The full versions of the selected documents were then reviewed by three independent reviewers. Of the studies evaluated, 57 were excluded for not meeting the inclusion criteria, leaving 101 articles for abstract review, and finally, 28 were included for full-text reading and the development of this review.

**Figure 1:**
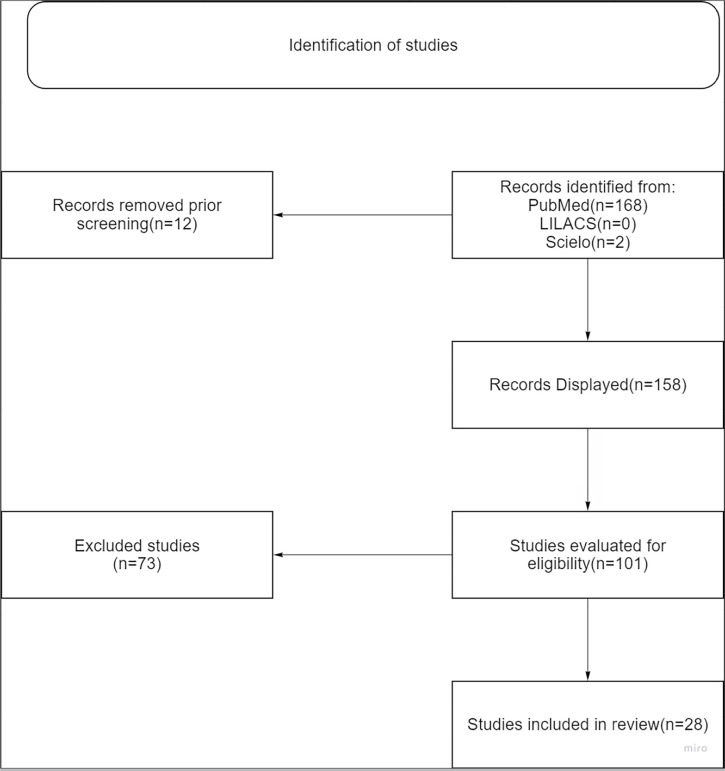
The flowchart of search and selection process. * Created by the authors.

The flowchart of search and selection process, so that it is clearer how the included articles were selected, is presented below on Figure 1. The type of each study chosen is presented in Table 1.

**Table 1:** The type of each study chosen was created by the authors.

Author(s)	Year	Study Title	Study Type	Findings by Topic
Almeida *et al*.	2018	Bisphenol A: Food Exposure and Impact on Human Health	Literature Review	Changes in Female and Male Fertility
Chen *et al*.	2013	Association of exposure to phenols and idiopathic male infertility	Cross-sectional Study	Changes in Female and Male Fertility
Chiang *et al*.	2017	Environmental Contaminants Affecting Fertility and Somatic Health	Literature Review	Changes in Female and Male Fertility
Czubacka *et al*.	2021	Urinary Bisphenol A Concentrations and Parameters of Ovarian Reserve	Observational Study	Changes in Female and Male Fertility
Eilam-Stock *et al*.	2012	Bisphenol-A impairs memory and reduces dendritic spine density	Experimental Study	Other Effects
Guo *et al*.	2023	Prenatal exposure to bisphenol A and neonatal health outcomes: A systematic review	Systematic Review	Gestational Changes
Knez	2013	Endocrine-disrupting chemicals and male reproductive health	Literature Review	Changes in Female and Male Fertility
Konieczna *et al*.	2015	Health risk of exposure to Bisphenol A (BPA)	Literature Review	Changes in Female and Male Fertility
Kundakovic *et al*.	2013	Sex-specific epigenetic disruption and behavioral changes	Experimental Study	Other Effects
Lü *et al*.	2024	Bisphenol A Exposure Interferes with Reproductive Hormones	Meta-analysis	Changes in Female and Male Fertility
Li *et al*.	2021	Physiologically detectable bisphenol A impairs human sperm functions	Experimental Study	Changes in Female and Male Fertility
Manfo *et al*.	2014	Adverse effects of bisphenol A on male reproductive function	Literature Review	Changes in Female and Male Fertility
Mansur *et al*.	2016	Does BPA alter steroid hormone synthesis in human granulosa cells?	Experimental Study	Changes in Female and Male Fertility
Matuszczak *et al*.	2019	The Impact of Bisphenol A on Fertility	Literature Review	Changes in Female and Male Fertility
Mínguez-Alarcón *et al*.	2015	Urinary bisphenol A concentrations and IVF outcomes	Observational Study	Assisted Reproduction
Pallotti *et al.*	2020	Mechanisms of Testicular Disruption from BPA and Phthalates	Literature Review	Changes in Female and Male Fertility
Pivonello *et al.*	2020	Bisphenol A: an emerging threat to female fertility	Literature Review	Changes in Female and Male Fertility
Rahman *et al.*	2015	Bisphenol-A affects male fertility via fertility-related proteins	Experimental Study	Changes in Female and Male Fertility
Rahman *et al.*	2017	Gestational exposure to BPA affects sperm function	Experimental Study	Gestational Changes
Rahman *et al.*	2018	Functional alterations of sperm following gestational BPA exposure	Experimental Study	Gestational Changes
Rochester & Bolden	2015	Bisphenol S and F: Comparison to BPA substitutes	Systematic Review	Changes in Female and Male Fertility
Sifakis *et al*.	2017	Human exposure to endocrine disrupting chemicals	Literature Review	Changes in Female and Male Fertility
Souter *et al*.	2013	Association of BPA urinary concentrations with ovarian reserve	Observational Study	Assisted Reproduction
Thacharodi *et al*.	2023	Endocrine disrupting chemicals and their effects on reproductive health	Literature Review	Changes in Female and Male Fertility
Vessa *et al*.	2022	Endocrine disruptors and female fertility	Literature Review	Changes in Female and Male Fertility
Zhang *et al*.	2023	Impacts of disinfection byproduct exposures on male reproductive health	Literature Review	Changes in Female and Male Fertility
Zhou *et al.*	2019	Association between prenatal exposure to bisphenol A and birth outcomes	Meta-analysis	Gestational Changes
Zlatnik	2016	Endocrine-disrupting chemicals and reproductive health	Literature Review	Changes in Female and Male Fertility

## LITERATURE REVIEW

The widespread use of BPA has raised concerns about its impact on human health, including reproductive health. This review examines data from recent literature to understand the implications of BPA exposure on reproductive health. The findings were separated by four topics: “changes in female and male fertility”, “assisted reproduction”, “gestational changes” and “other effects”.

### Changes in female and male fertility

In recent years, more studies have been published on the association between BPA and its analogs with male and female fertility, widely linking it to various adverse effects, including the disruption of hormonal balance, negative impacts on ovarian function, and potentially compromised fertility. A recently published systematic review and meta-analysis consistently found an association between BPA exposure and reproductive disorders, attributed to BPA’s ability to mimic estrogen and interfere with ovarian function, thereby affecting fertility (Lü *et al.*, 2024). Additionally, provided experimental evidence that even low levels of BPA exposure can lead to reproductive cellular toxicity, highlighting its detrimental effects. BPA interacts with estrogen receptors and can act as both an agonist and antagonist through endocrine receptor-dependent signaling pathways, contributing to the pathogenesis of various endocrine disorders, early puberty, and hormone-dependent tumors, such as breast and prostate cancer. The occurrence of polycystic ovary syndrome (PCOS) has also been linked to BPA exposure (Chen *et al.*, 2013; Konieczna *et al.*, 2015; Sifakis *et al.*, 2017; Vessa *et al.*, 2022; Thacharodi *et al.*, 2023).

Although the exact mechanism by which BPA interferes with fertility is still unclear, studies have shown that BPA levels may be associated with decreased antral follicle counts and reduced oocyte counts (Konieczna *et al.*, 2015; Sifakis *et al.*, 2017). Furthermore, animal models exposed to BPA have reported the occurrence of endometriosis-like lesions (Pivonello *et al.*, 2020). During the postnatal and pubertal periods and into adulthood, BPA seems to affect the hypothalamic-pituitary-testicular axis by modulating hormone synthesis (e.g., LH, FSH, androgens, and estrogens), as well as the expression and function of their respective receptors. These effects alter sperm parameters (Zhang *et al.*, 2023). BPA also may induce oxidative stress in the testes and epididymis by inhibiting antioxidant enzymes and promoting lipid peroxidation. This suggests that the use of antioxidants may be a promising strategy to mitigate BPA-induced disorders (Zhang *et al.*, 2023). Additionally, men exposed to BPA have shown reduced libido, retrograde ejaculation, and erectile dysfunction (Zhang *et al.*, 2023).

Furthermore, studies demonstrate alterations in protein profiles related to fertility in spermatozoa following exposure to BPA, and these changes appear to be associated with a decline in fertilization rates and embryonic development (Rahman *et al.*, 2015; 2017; 2018). Several authors of the analyzed studies have drawn a parallel between BPA exposure and sperm quality. For example, some researchers investigated urinary phenols and semen parameters in a large case-control study, comparing idiopathically infertile men with fertile controls. They found a significant association between alkylphenols and idiopathic male infertility with abnormal seminal parameters, but this association was not found with other phenols included in the analysis, only with BPA (Chen *et al.*, 2013). Another study also demonstrated that physiologically detectable concentrations of BPA can impair sperm function by suppressing protein tyrosine phosphorylation (Li *et al.*, 2021).

Studies conducted by Chen *et al.* (2013) reveal that BPA, at concentrations greater than 10⁻^6^M, inhibits the proliferation of Leydig cells. It was found that 50 proteins were modulated in these cells after exposure to BPA. Significant effects were observed on the structure, motility, cellular metabolism, protein and nucleotide processing, and cellular proliferation of these cells.

The study also demonstrated that, at micromolar concentrations or higher, BPA can modulate protein profiles, inhibit cell proliferation, and promote the in vitro migration and invasion of Leydig TM3 cells, a Leydig cell that was isolated from a male mouse, which can be explained by the modulation of proteins related to cell structure and motility, such as actin and heat shock proteins (Chen *et al.*, 2013).

There is chemical’s potential role in reduced sperm count and motility and, corroborating these findings, a recently published study concluded that BPA exposure may negatively impact male fertility, highlighting reduced sperm quality and altered hormone levels in men (Zhang *et al.*, 2023). Besides, BPA exposure impairment on male fertility is associated with endocrine-disrupting chemicals but its exact hole is still under investigation (Zlatnik, 2016).

Hence, considering both male and female reproductive health when assessing BPA’s overall impact is important to fully understand how it affects both genders. There is an urge for more research to fully understand the extent of BPA’s impact on reproductive human health, given the variability of studies’ findings (Zlatnik, 2016).

### Assisted Reproduction

Studies have shown an association between BPA and a worsened ovarian response, a lower number of mature oocytes collected and fertilized, and a reduced concentration of estradiol (E2) in response to hyperstimulation with gonadotropin. This was further associated with lower fertilization rates and increased implantation failure. Embryo quality in women undergoing IVF treatment was also affected (Sifakis *et al.*, 2017). It is believed that exposure to high concentrations of BPA may increase the apoptosis of cells that play a crucial role in oocyte protection. However, limited information is available regarding the effects of BPA exposure in adults on ovarian reserve (Chiang *et al.*, 2017; Almeida *et al.*, 2018), and the studies remain controversial. For instance, Mínguez-Alarcón *et al.* (2015) found that urinary BPA concentrations were not associated with endometrial wall thickness, peak estradiol levels, the proportion of high-quality embryos, or fertilization rates. Additionally, no association was found between urinary BPA concentrations and implantation, clinical pregnancy, or live birth rates per cycle initiated or per embryo transfer (Mínguez-Alarcón *et al.*, 2015).

However, urinary BPA was detected in the majority of samples collectewd from women seeking infertility treatments (Souter *et al.*, 2013). These findings support in vitro results and animal studies, highlighting the potential risk of BPA exposure to human reproductive health.

Mansur *et al.* (2016) demonstrated that treating luteinized granulosa cells with BPA resulted in a significant decrease in estradiol and progesterone biosynthesis, which was confirmed by a notable reduction in mRNA and protein expression levels of three genes encoding steroidogenesis enzymes: CYP11A1, 3β-HSD, and CYP19A1. However, this effect was observed only at very high, supra-physiological levels. The results suggest that BPA levels detected in blood, urine, and ovarian follicles do not negatively affect granulosa cell steroidogenesis in vitro. These conclusions may be reassuring for women trying to conceive, given the ubiquity of BPA exposure (Mansur *et al.*, 2016).

### Uterine changes

Using animal models (rats), it was found that low doses of BPA affect uterine development, leading to fewer implantation sites and various changes in the endocrine pathways that regulate endometrial preparation for embryo implantation. The study reported that early changes in the expression of the HOXA10 gene disrupt the functional differentiation of the uterus during pregnancy, as part of an altered endocrine signal transduction pathway (Zhou *et al.*, 2019).

### Gestational changes

The impact of BPA on pregnancy outcomes is a critical area of concern, as BPA exposure has been associated with an increased risk of miscarriage, preterm birth, and other adverse pregnancy outcomes (Li *et al.*, 2021). The authors confirmed these findings through a meta-analysis showing a significant association between BPA exposure and miscarriage risk.

Additionally, BPA exposure may potentially alter endometrial function and impact fetal development at various stages of pregnancy, suggesting that the effects of this chemical extend beyond conception to include gestation and birth outcomes (Guo *et al.*, 2023).

Maternal exposure to BPA during fetal development presents a greater risk to the developing organism. Exposure during this phase is associated with disrupted testosterone production and reproductive tract development, as demonstrated by in vivo and in vitro detection of multiple antiandrogen endocrine disruptors. Fetal life is a critical phase when sex is determined by the differentiation of reproductive tissues-a fundamental step for the future development and maintenance of reproductive function. In the male fetus, these processes rely on the initiation and maintenance of androgenic activity (Pallotti *et al.*, 2020).

Such processes, however, can be disrupted by endocrine disruptors at various levels, including androgen receptor (AR) antagonism and interference in steroid hormone synthesis. Disruption of these processes can result in damage to reproductive tissues while the fetus is still in the womb, leading to malformations such as hypospadias, cryptorchidism, testicular hypertrophy, reduced anogenital distance, impaired future fertility, altered spermatogenesis, infertility, and the development of testicular tumors (Pallotti *et al.*, 2020). BPA may be associated with several defects in the embryo, such as feminization of male fetuses, atrophy of the testicles and epididymis, increased prostate size, and alterations in adult sperm parameters (e.g., sperm count, motility, and density), and also may affect the development of the embryonic thyroid (Manfo *et al.*, 2014).

Various endocrine disruptors, including BPA, are still widely detected in the environment, and the quantity of these substances that would be harmful to health remains unknown. The period of sexual differentiation appears to be particularly susceptible to the negative effects of environmental pollutants-exposure to phthalates, for example, has been linked to a higher incidence of developmental anomalies (Knez, 2013).

BPA exposure during pregnancy also affects brain development, potentially inducing anxiety, an increased risk of autistic behaviors, impaired memory and learning, as well as changes in social behaviors (Almeida *et al.*, 2018). Moreover, BPA exposure induces specific gender-related alterations, with dose-dependent and brain-region-dependent expression of genes encoding estrogen receptors (ERs, ERα, ERβ, and ER) (Almeida *et al.*, 2018). Additionally, the formation of new memories may be affected, as BPA appears to interfere with neuronal plasticity processes. BPA reduces synaptic density, increases the length of the synaptic cleft, reduces the length of the active zone, and decreases postsynaptic density in the hippocampus (Eilam-Stock *et al.*, 2012; Kundakovic *et al.*, 2013; Almeida *et al.*, 2018). It is important to note that interpreting the results of these studies, especially those related to behavior, can be challenging, as some inherent factors and variables may distort the conclusions. Furthermore, a longitudinal study found that BPA concentrations in maternal urine were associated with increased anxiety and depression in boys, but not in girls, at seven years of age (Almeida *et al.*, 2018).

In a random-effects meta-analysis of prenatal BPA exposure and hyperactivity showed that early BPA exposure in humans was associated with hyperactivity in both boys and girls. One explanation for BPA’s interference with the central nervous system is its association with a decrease in total thyroxine in pregnant women and in thyroid-stimulating hormone (TSH) in newborn males (Rochester & Bolden, 2015).

Additionally, prenatal low-dose BPA exposure in a rat model showed changes in thyroid receptor expression in the fetal neocortex. This evidence suggests that perinatal hypothyroxinemia caused by BPA exposure during pregnancy may lead to neurological deficits (Almeida *et al.*, 2018). The dopamine system is also affected by early BPA exposure, which may explain certain behaviors, such as anxiety. The number of dopamine neurons appears to decrease in the midbrain, along with interference in the activity of tyrosine hydroxylase, the rate-limiting enzyme for dopamine synthesis (Almeida *et al.*, 2018).

## CONCLUSION

The present study aimed to evaluate the possible association of BPA with human reproductive function. The reviewed literature consistently indicates that exposure to BPA has significant implications for reproductive health in both males and females, with evidence suggesting risks even at low doses. Current findings highlight BPA’s role as an endocrine disruptor that can interfere with hormonal balance, ovarian function, and pregnancy outcomes. While the included studies provide a solid foundation, further human research is necessary to fully understand the mechanisms of these effects, the potential long-term consequences, and how to develop strategies to mitigate these risks through assisted reproductive technologies.

### Limitations of the study

Even though the extensive review of available literature on the effects of Bisphenol A (BPA) on reproductive health, several limitations should be considered when interpreting the results of this study. First of all, the majority of the included studies are observational or experimental, which often carry inherent biases and limitations in establishing causality. Many of the studies rely on self-reported data for exposure assessment, which may be subject to recall bias and inaccuracies. Furthermore, variations in study design, such as differences in sample size, population demographics, and exposure levels, may affect the generalizability of the findings. In addition, there is a lack of standardized protocols for measuring BPA exposure, making it difficult to compare results across studies. Moreover, the long-term effects of BPA exposure, especially at low doses, remain uncertain due to the absence of large, long-term cohort studies. Subsequently, while this review highlights significant associations between BPA exposure and various reproductive health outcomes, it is important to recognize that BPA is just one of many environmental endocrine-disrupting chemicals, and future studies should explore the combined effects of multiple exposures.

## References

[r1] Almeida S, Raposo A, Almeida-González M, Carrascosa C. (2018). Bisphenol A: Food Exposure and Impact on Human Health. Compr Rev Food Sci Food Saf.

[r2] Chen M, Tang R, Fu G, Xu B, Zhu P, Qiao S, Chen X, Xu B, Qin Y, Lu C, Hang B, Xia Y, Wang X. (2013). Association of exposure to phenols and idiopathic male infertility. J Hazard Mater.

[r3] Chiang C, Mahalingam S, Flaws JA. (2017). Environmental Contaminants Affecting Fertility and Somatic Health. Semin Reprod Med.

[r4] Czubacka E, Wielgomas B, Klimowska A, Radwan M, Radwan P, Karwacka A, Kałużny P, Jurewicz J. (2021). Urinary Bisphenol A Concentrations and Parameters of Ovarian Reserve among Women from a Fertility Clinic. Int J Environ Res Public Health.

[r5] Eilam-Stock T, Serrano P, Frankfurt M, Luine V. (2012). Bisphenol-A impairs memory and reduces dendritic spine density in adult male rats. Behav Neurosci.

[r6] Guo J, Liu K, Yang J, Su Y. (2023). Prenatal exposure to bisphenol A and neonatal health outcomes: A systematic review. Environ Pollut.

[r7] Knez J. (2013). Endocrine-disrupting chemicals and male reproductive health. Reprod Biomed Online.

[r8] Konieczna A, Rutkowska A, Rachoń D. (2015). Health risk of exposure to Bisphenol A (BPA). Rocz Panstw Zakl Hig.

[r9] Kundakovic M, Gudsnuk K, Franks B, Madrid J, Miller RL, Perera FP, Champagne FA. (2013). Sex-specific epigenetic disruption and behavioral changes following low-dose in utero bisphenol A exposure. Proc Natl Acad Sci U S A.

[r10] Li N, Kang H, Peng Z, Wang HF, Weng SQ, Zeng XH. (2021). Physiologically detectable bisphenol A impairs human sperm functions by reducing protein-tyrosine phosphorylation. Ecotoxicol Environ Saf.

[r11] Lü L, Liu Y, Yang Y, He J, Luo L, Chen S, Xing H. (2024). Bisphenol A Exposure Interferes with Reproductive Hormones and Decreases Sperm Counts: A Systematic Review and Meta-Analysis of Epidemiological Studies. Toxics.

[r12] Manfo FP, Jubendradass R, Nantia EA, Moundipa PF, Mathur PP. (2014). Adverse effects of bisphenol A on male reproductive function. Rev Environ Contam Toxicol.

[r13] Mansur A, Adir M, Yerushalmi G, Hourvitz A, Gitman H, Yung Y, Orvieto R, Machtinger R. (2016). Does BPA alter steroid hormone synthesis in human granulosa cells in vitro?. Hum Reprod.

[r14] Matuszczak E, Komarowska MD, Debek W, Hermanowicz A. (2019). The Impact of Bisphenol A on Fertility, Reproductive System, and Development: A Review of the Literature. Int J Endocrinol.

[r15] Mínguez-Alarcón L, Gaskins AJ, Chiu YH, Williams PL, Ehrlich S, Chavarro JE, Petrozza JC, Ford JB, Calafat AM, Hauser R, EARTH Study Team (2015). Urinary bisphenol A concentrations and association with in vitro fertilization outcomes among women from a fertility clinic. Hum Reprod.

[r16] Pallotti F, Pelloni M, Gianfrilli D, Lenzi A, Lombardo F, Paoli D. (2020). Mechanisms of Testicular Disruption from Exposure to Bisphenol A and Phtalates. J Clin Med.

[r17] Pivonello C, Muscogiuri G, Nardone A, Garifalos F, Provvisiero DP, Verde N, de Angelis C, Conforti A, Piscopo M, Auriemma RS, Colao A, Pivonello R. (2020). Bisphenol A: an emerging threat to female fertility. Reprod Biol Endocrinol.

[r18] Rahman MS, Kwon WS, Karmakar PC, Yoon SJ, Ryu BY, Pang MG. (2017). Gestational Exposure to Bisphenol A Affects the Function and Proteome Profile of F1 Spermatozoa in Adult Mice. Environ Health Perspect.

[r19] Rahman MS, Kwon WS, Lee JS, Yoon SJ, Ryu BY, Pang MG. (2015). Bisphenol-A affects male fertility via fertility-related proteins in spermatozoa. Sci Rep.

[r20] Rahman MS, Kwon WS, Ryu DY, Khatun A, Karmakar PC, Ryu BY, Pang MG. (2018). Functional and Proteomic Alterations of F1 Capacitated Spermatozoa of Adult Mice Following Gestational Exposure to Bisphenol A. J Proteome Res.

[r21] Rochester JR, Bolden AL. (2015). Bisphenol S and F: A Systematic Review and Comparison of the Hormonal Activity of Bisphenol A Substitutes. Environ Health Perspect.

[r22] Sifakis S, Androutsopoulos VP, Tsatsakis AM, Spandidos DA. (2017). Human exposure to endocrine disrupting chemicals: effects on the male and female reproductive systems. Environ Toxicol Pharmacol.

[r23] Souter I, Smith KW, Dimitriadis I, Ehrlich S, Williams PL, Calafat AM, Hauser R. (2013). The association of bisphenol-A urinary concentrations with antral follicle counts and other measures of ovarian reserve in women undergoing infertility treatments. Reprod Toxicol.

[r24] Thacharodi A, Hassan S, Acharya G, Vithlani A, Hoang Le Q, Pugazhendhi A. (2023). Endocrine disrupting chemicals and their effects on the reproductive health in men. Environ Res.

[r25] Vessa B, Perlman B, McGovern PG, Morelli SS. (2022). Endocrine disruptors and female fertility: a review of pesticide and plasticizer effects. F S Rep.

[r26] Zhang M, Deng YL, Liu C, Lu WQ, Zeng Q. (2023). Impacts of disinfection byproduct exposures on male reproductive health: Current evidence, possible mechanisms and future needs. Chemosphere.

[r27] Zhou Z, Lei Y, Wei W, Zhao Y, Jiang Y, Wang N, Li X, Chen X. (2019). Association between prenatal exposure to bisphenol a and birth outcomes: A systematic review with meta-analysis. Medicine (Baltimore).

[r28] Zlatnik MG. (2016). Endocrine-Disrupting Chemicals and Reproductive Health. J Midwifery Womens Health.

